# Mental health inequities affecting sexual and gender diverse individuals during the early COVID-19 period in Massachusetts

**DOI:** 10.1371/journal.pmen.0000341

**Published:** 2025-12-19

**Authors:** Jessica H. Leibler, Yirong Yuan, Elizabeth Beatriz, Ta-wei Lin, McKane Sharff, Caroline Stack, Lauren Cardoso, Koen Tieskens, Kerra Washington, Xiaojing Peng, Prasad Patil

**Affiliations:** 1 Department of Environmental Health, Boston University School of Public Health, Boston, Massachusetts, United States of America; 2 Department of Biostatistics, Boston University School of Public Health, Boston, Massachusetts, United States of America; 3 Office of Statistics and Evaluation, Bureau of Community Health and Prevention, Massachusetts Department of Public Health, Boston, Massachusetts, United States of America; Liverpool John Moores University, UNITED KINGDOM OF GREAT BRITAIN AND NORTHERN IRELAND

## Abstract

While the COVID-19 pandemic negatively affected population-level mental health, research on mental health among sexual and gender minority (SGM) individuals and intersectional identities is limited. We evaluated associations between gender, sexual, racial, and ethnic identities and moderate to severe psychological distress (≥15 days of self-reported poor mental health during the last 30 days) among adult residents of Massachusetts (n = 26,889) from September-November 2020. We used multivariable logistic regression with propensity score weighting and considered intersectional identities by race, ethnicity, sexual orientation and gender. Results: Respondents identifying as lesbian, gay, bisexual, queer, asexual, or questioning (LGB+) experienced 83% increased odds of psychological distress compared to heterosexual/straight individuals (OR: 1.83 (1.34, 2.50); p < 0.001). Respondents identifying as transgender and/or nonbinary (TNB) had the highest prevalence of psychological distress and experienced 57% increased odds of psychological distress compared to the referent group (OR: 1.57 (1.21, 2.04); p < 0.001). Respondents identifying as Hispanic/Latinx, Black non-Hispanic (NH), or Asian NH reported reduced odds of psychological distress (Hispanic (OR: 0.75 (0.67, 0.84); p < 0.001), Black NH (OR: 0.75 (0.64, 0.88); p < 0.001), and Asian NH (OR: 0.61 (0.51,0.73); p < 0.001). We observed reduced odds of psychological distress among LGB+ respondents of color (OR: 1.03 (0.82, 1.29); p = 0.83) and TNB respondents of color (OR: 1.27 (0.73, 2.20); p = 0.39) compared to LGB+ and TNB estimates alone, although neither interaction was statistically significant. Mental health inequities among SGM individuals in Massachusetts were exacerbated during the early pandemic period. Social and structural inequities experienced by SGM individuals may contribute to higher risk of psychological distress. Public health policies and practices to address the root causes of inequities and promote mental health among SGM communities are warranted.

## Introduction

The COVID-19 pandemic had devastating consequences for mental health around the world [1,2]. These impacts stem from morbidity and mortality associated with COVID-19 infections as well as changes to employment, education, childcare, eldercare, and other aspects of daily life. Pre-existing social and structural vulnerabilities and health inequities, as well as elevated exposure to pandemic-induced stressors, including job loss, loss of care providers or support services, and reduced access to in-person healthcare services, resulted in disproportionate mental health impacts borne by marginalized groups, with implications for sustained or exacerbated disparities [3,4]. Despite this, marginalized groups are often underrepresented in population-level research that informs policy and resource allocation.

Individuals who are gender-diverse and with sexual orientations other than heterosexual (termed here sexual and gender minorities, SGM) often face unique challenges that can negatively impact mental health, stemming from intertwined social, cultural, and systemic factors [5–7]. For example, stigma and discrimination against SGM individuals can cause and exacerbate mental health challenges [8]. During the COVID-19 pandemic, some transgender individuals encountered disruptions in accessing gender-affirming healthcare, due to overwhelmed healthcare systems and restrictions on medical procedures deemed non-essential [9–11]. Research to date on the mental health impacts of COVID-19 on SGM populations suggest that reduced social and family supports experienced by these individuals may have contributed to worsened mental health burdens, specifically around depression, anxiety, disordered eating, suicidal thoughts, self-harm, and substance abuse [12,13]. Despite these established stressors, there are limited population-level studies to date of SGM mental health experiences during the pandemic, often because detailed data on these identities (beyond a male/female binary or grouping of sexual and gender identities) are not collected [7].

These disparities are well described within the minority stress framework, which suggests that chronic exposure to stigma, discrimination, and structural marginalization generates sustained psychological stress and elevated risk of adverse mental health outcomes among sexual and gender minority individuals [14,15]. This framework emphasizes that both structural and internalized stigma can interact with broader societal stressors combine to compound vulnerability to psychological distress.

A growing literature depicts mental health disparities associated with SGM identity during the COVID-19 pandemic [16–18]. Notably, these findings cut across occupational and socioeconomic groups, cultures and regions, and extend from early to late pandemic periods. Breslow and colleagues identified elevated rates of depression, anxiety and psychological distress among SGM frontline medical workers during the early pandemic months [19]. In a similar study to CCIS in Canada, investigators noted particular poor mental health outcomes associated with bisexual and asexual identities [6]. The authors noted that social support had a stronger modifying effect on depression risk among SGM compared to cisgender heterosexual respondents, highlighting the value of social support for these groups in particular. Other analyses found higher risk of substance abuse, suicidal thoughts and self-harm among SGM individuals, suggesting the value of improved social supports for SGM communities during periods of challenge or social isolation [20–22].

Additionally, there is a growing literature detailing pandemic-induced health inequities in access to care, disease severity, and community-level recovery among communities of color. In Massachusetts, we and others observed striking racial and ethnic disparities in COVID-19 case burden and mortality during the first year of the pandemic [23,24]. Racial and ethnic disparities are also observed in vaccination uptake and in unemployment rates [25,26]. Research to date on mental health inequities indicates worsening mental health outcomes among individuals of color, due in part to concurrent social stress resulting from racism and discrimination [3,27]. The impacts of the pandemic on SGM individuals of color remains less studied. Larger, population-level studies that capture multiple non-majority identities can provide a more comprehensive understanding of the mental health burden associated with the pandemic, identify groups inequitably impacted, and prioritize public health resources to communities that are inequitably burdened.

In the fall of 2020, the Massachusetts Department of Public Health (MDPH), in collaboration with community and academic partners, initiated the COVID-19 Community Impact Survey (CCIS) to identify the needs of resident populations most impacted by the pandemic [28,29]. The study’s goals included identifying inequities across physical and mental health, economic impact, access to resources, and overall well-being to inform targeted support and intervention strategies. Massachusetts represents a particularly relevant case study given its substantial early pandemic case burden, high levels of urban population density, and relatively progressive policy environment regarding SGM rights and health equity [23,30,31]. (Figueroa et al., 2020; Movement Advancement Project, 2023). Examining this setting provides insight into how social and structural inequities persisted even in a state with strong public health infrastructure and inclusive legal protections. The analysis here using CCIS data reflects a collaboration between MDPH and researchers at Boston University School of Public Health focused on applying statistical tools to evaluating health inequities created and exacerbated by the pandemic [32,33].

We hypothesized that we would observe disproportionately poor mental health during the early pandemic period among SGM compared to heterosexual cisgender individuals and those identifying as Hispanic/Latinx, Black non-Hispanic (NH), or Asian NH compared to those identifying as White in Massachusetts. Secondly, we hypothesized that individuals with multiple marginalized identities among these groups – notably SGM individuals of color - may experience worse mental health endpoints than those without such intersectional identities.

## Materials and methods

### CCIS survey

CCIS was conducted anonymously from September 17 to November 20, 2020,as an online survey. The survey was available in 11 languages, with different versions for young people (14–24 years) and adults (≥25 years). A core intention of the survey was to gather data from individuals who are typically underrepresented in traditional public health data sources, including SGM and people of color, to inform public health action in the state during the pandemic [29]. Recruitment was conducted in partnership with community-based organizations using a snowball sampling approach, with a focus on key populations, as well as through traditional outreach approaches. Demographic data included sexual orientation and gender identity, transgender experience, race, ethnicity, and age. Questions on CCIS related to these topics were derived using MDPH data standards (Table 1). Our analysis focused on the adult subset only for methodological standardization and generalizability issues. All study protocols involving human subjects were approved by the MDPH IRB (protocol #1605581). Participants provided informed consent through an online consent form. Participation in CCIS was anonymous and individual identifiers (gender, sexuality, race, ethnicity) were self-reported. Statistical analyses used in this manuscript were exempted from review by the IRB at Boston University Medical Center because the data shared with BU investigators were de-identified.

**Table 1:** 2020 CCIS Demographic Question, Response Options and Groupings (n = 26,889).

Demographic	Survey Responses (n)	Model 1	Model 2
Sexual Orientation	What is your sexual orientation? **Straight (Heterosexual) (22,905)** **LGB+ (3,193):** Asexual (471) Bisexual and/or Pansexual (1,078) Gay or Lesbian (1,166) Queer (415) Other, (please specify) (63) Questioning/I am not sure of my sexuality (178) I don’t understand what this question is asking (393)	**Straight/ Heterosexual (Ref)** **LGB+ (all combined)** Excluded Excluded	**Straight/ Heterosexual (Ref)** Bisexual and/or Pansexual Other (Asexual, Queer, Other) Excluded Excluded
Gender Identity	*What is your current gender identity?* **Cisgender (26,466)** Male (5,295) Female (21,257) **Not cisgender (407):** Nonbinary, Genderqueer, not exclusively male or female (276) I am questioning/not sure of my gender identity (48) Other (please specify:) (8) I don’t understand what this question is asking (1) I prefer not to answer (4)	**Cisgender = 0 (ref)** Excluded transgender (below) **Not cisgender = 1 (all combined)** Included transgender (below) Excluded Excluded	Male (ref) Female Non-binary/Genderqueer Other Excluded Excluded
Transgender Identity	*Are you transgender or of transgender experience?* **No (26,669):** No (26,566) I’m not sure (83) I don’t understand what this question is asking (7) I prefer not to answer (13) **Yes (217)**	**No = 0 (all combined) (ref)** **Yes = 1**	**No = 0 (all combined) (ref)** **Yes = 1**
Race and Hispanic/Latinx Ethnicity	*Are you Hispanic or Latino?* **No (25,016)** **Yes (1,873)**	**No = 0 (ref)** **Yes = 1**	**No = 0 (ref)** **Yes = 1**
	*What is your race? Select all that apply.* **White (22,492)** **Person of color (4,397):** American Indian or Alaska Native (please specify tribal nation) (265) Asian (874) Black (859) Multiracial (376) Other Race (213) Unknown/ not specified (334)	**White = 0 (ref)** **Person of color = 1 (all combined)** Included Hispanic/Latino (above) Excluded	**White = 0 (ref)** American Indian or Alaska Native, Asian, Black, Hispanic/Latino, Multiracial, Other Excluded

### Psychological distress

We estimated experience of psychological distress using the following question, which is a component of a CDC-validated set of questions called the “Healthy Days” series: “Now thinking about your mental health, which includes stress, depression, and problems with emotions, for how many days during the past 30 days was your mental health not good?” The Healthy Days series is validated in individuals 12 years and older and is a long-standing component of the BRFSS and the National Health and Nutrition Examination Survey (NHANES) [34,35]. Psychological distress was evaluated dichotomously, using 15 or more days of reported poor mental health as a threshold for moderate or severe psychological distress, per CDC [36].

### Data analysis

CCIS respondents were included in the analysis if they reported living in Massachusetts, responded to demographic questions regarding age, race, gender, and education, and answered the “Mental Health Days” question. Individuals who did not provide responses to demographic questions or did not answer the outcome question were excluded from analysis; we did not impute missing demographic or outcome data. CCIS respondents were disproportionately women, college-educated and higher income compared to MA statewide estimates. To address this imbalance, we used propensity score weighting to generate survey weights for the CCIS dataset using the US Census Bureau’s Public Use Microdata Sample (PUMS) from Massachusetts as a referent. The PUMS provides individual-level data from the American Community Survey at 1- and 5-year increments [37]. These weights were used to align the distribution of relevant covariates in the CCIS dataset with this reference sample with a single weight generated for each individual, reflecting their probability of being in the CCIS sample compared to the PUMS sample. We generated weights with logistic regression using age, gender, race, and education as covariates and a 0/1 indicator of whether an observation is from the CCIS or PUMS survey as the outcome. The final weights were the inverse predicted probability of belonging to the CCIS dataset. To note: CCIS and PUMS collect data on gender using different categories. PUMS uses a dichotomous male [1] and female [2] variable, while CCIS includes multiple categories (Table 1). To account for this issue, we fitted an additional logistic regression model balancing only on age, race, and education, excluding gender from this approach. Individuals identifying as nonbinary or other/unsure were assigned weights using this second model. All procedures were implemented using R version 4.2.2 (R Core Team, 2022) and the *NonProbEst* R package [38]. See S1 Fig for a diagram of the propensity score modeling process.

### Regression modeling

We used adjusted multivariable logistic regression with the dichotomous dependent variable of ≥15 days of poor mental health as reflective of moderate or severe psychological distress [36]. We adjusted for covariates that we hypothesized *a priori* may be associated with mental health in the fall of 2020, including town-level COVID-19 transmission at the time of survey completion (dichotomized as above or below the median case burden during the study period), household income (<$35,000, $35,000-$100,000, and>$100,000 annually), age (25–99), region within the state, and employment status (employed, unemployed, retired). Covariates were selected based on theoretical relevance and prior literature, focusing on identity and contextual factors (e.g., race/ethnicity, sexual orientation, transgender experience, COVID-19 transmission, socioeconomic indicators). Town-level COVID-19 transmission data from the fall of 2020 was generated from publicly available MDPH dashboards [39]. Region of residence was extracted from self-reported town of residence on the survey and divided into four geographic regions (Northeast, including Boston and the Boston Metrowest area; Southeast; Central; and Western MA). We developed two models, the first of which (Model 1) incorporated dichotomous variables to consider sexual orientation, gender identity, and race/Hispanic ethnicity characteristics as depicted in Table 1: alongside the covariates above. In Model 1, we generated interaction terms to reflect pairwise intersections between sexual orientation and gender identity variables, race/Hispanic ethnicity, and select covariates; categories were combined to increase power and test selected interactions. In Model 2, we disaggregated the identity variables to consider more nuanced categorization of sexual orientation, gender, and race/ethnicity identifications (Table 1). We did not evaluate intersections in Model 2 due to small counts in some identity categories. We reduced multicollinearity, combined categories as justified to increase power and tested selected interactions.

## Results

Overall, 33,800 adults aged 25 years and older participated in the CCIS study. Per our inclusion criteria, respondents who did not complete the mental health days question were excluded from the analysis (n = 5,900). Among the remaining respondents, we additionally excluded 1,011 respondents for whom analytic survey weights could not be constructed due to missing responses to demographic variables (race, gender, age, or education). In total, 26,889 respondents were included in this analysis. Respondents predominantly identified as female (79.1%), with 19.7% identifying as male and 1.2% identifying as non-binary. Additionally, 0.8% identified as being of transgender experience. For sexual orientation, 85.2% or respondents identified as heterosexual, 4.3% as lesbian or gay, 4.0% as bisexual and 3.5% as some other sexual orientation. Considering racial and Hispanic ethnic identities, 3.2% identified as Black NH, 6.7% as Hispanic/Latinx, 3.3% as Asian NH, and 83.6% as White NH (Table 2, 3).

**Table 2:** Prevalence and selected identities and frequent psychological distress among Massachusetts residents, Fall 2020 (n = 26,889).

	n (% of total sample)	Prevalence of frequent psychological distress % (n)^1^
**Total**	26,889 (100)	33.3 (8,591)
**Gender identity**		
Model 1:^1^		
Cisgender (ref)	26,446 (98.4)	31.4 (8,315)
Not cisgender	407 (1.5)	63.6 (259)
Model 2:		
Male (ref)	5,295 (19.7)	24.4 (1,290)
Female	21,257 (79.1)	33.3 (7,082)
Nonbinary, genderqueer, or not male or female	332 (1.2)	64.8 (215)
**Transgender experience**		
Not transgender (ref)	26,669 (99.2)	31.7 (8,458)
Transgender	217 (0.8)	60.4 (131)
**Sexual orientation**		
Model 1:		
Heterosexual/straight (ref)	22,905 (85.2)	29.9 (6,847)
Lesbian, gay, bisexual, or other non-heterosexual identity	3,193 (11.9)	45.5 (1,452)
Model 2:		
Heterosexual (ref)	22,905 (85.2)	29.9 (6,847)
Lesbian or gay	1,166 (4.3)	37.0 (432)
Bisexual	1,078 (4.0)	51.9 (559)
Other sexual orientation	949 (3.5)	48.6 (461)
**Race and ethnicity**		
Model 1:		
White (ref)	22,492 (83.6)	31.8 (7,147)
People of color	4,397 (16.4)	32.8 (1,444)
Model 2:		
White (ref)	22,492 (83.6)	31.8 (7,147)
Black NH	859 (3.2)	32.5 (279)
Hispanic/Latino	1,810 (6.7)	34.4 (622)
Asian NH	874 (3.3)	24.1 (211)
American Indian/Alaska Native	265 (1.0)	40.4 (107)
Multiracial NH	376 (1.4)	40.2 (151)
Other Race NH	213 (0.8)	34.7 (74)

^1^Model 1 aggregates identity variables into dichotomous categories and Model 2 uses more detailed identity responses. Gender covariates in Model 1 are defined as cisgender (male or female AND not of transgender experience) or not cisgender (transgender experience, non-binary, genderqueer, not exclusively male or female; I am questioning/not sure of my gender identity; Other AND/OR reporting transgender experience. For Model 2, we characterize self-reported gender.

**Table 3:** Adjusted odds ratios (95% CI) of frequent psychological distress by selected identities among Massachusetts residents, Fall 2020 (n = 26,889).

Gender identity	Adjusted odds ratio (OR; 95%CI)^1^
Model 1:^2^	
Cisgender (ref)	–
Not cisgender	1.57 (1.21, 2.04)
Model 2:	
Male (ref)	–
Female	1.46 (1.36, 1.58)
Nonbinary, genderqueer, or not male or female	1.73 (1.23, 2.44)
**Transgender experience**	
Not transgender (ref)	–
Transgender	1.32 (0.92, 1.91)
**Sexual orientation**	
Model 1:	
Heterosexual/straight (ref)	–
Lesbian, gay, bisexual, or other non-heterosexual identity^3^	1.83 (1.34, 2.50)
Model 2:	
Heterosexual (ref)	–
Lesbian or gay	1.44 (1.26, 1.64)
Bisexual	1.74 (1.52, 1.99)
Other sexual orientation^3^	1.56 (1.34, 1.82)
**Race and ethnicity**	
Model 1:	
White (ref)	–
People of color	0.68 (0.51, 0.91)
Model 2:	
White (ref)	–
Black NH	0.75 (0.63, 0.88)
Hispanic/Latino	0.75 (0.67, 0.84)
Asian NH	0.61 (0.51, 0.73)
American Indian/Alaska Native	1.18 (0.89, 1.56)
Multiracial NH	1.11 (0.88, 1.40)
Other Race NH	1.14 (0.83, 1.57)

^1^Adjusted ORs generated from multivariable logistic regression models. Covariates in both Models 1 and 2 included town-level COVID-19 transmission at the time of survey completion, household income, age, region, and employment status.

^2^Model 1 aggregates identity variables into dichotomous categories and Model 2 uses more detailed identity responses. Gender covariates in Model 1 are defined as cisgender (male or female AND not of transgender experience) or not cisgender (transgender experience, non-binary, genderqueer, not exclusively male or female; I am questioning/not sure of my gender identity; Other AND/OR reporting transgender experience. For Model 2, we characterize self-reported gender.

^3^Sexual orientation in Model 1 is depicted as a dichotomous variable as heterosexual/straight as the referent population and the non-heterosexual identities as noted as the positive class. “Other sexual orientation” includes queer, asexual, questioning, and other. These responses were aggregated due to small counts to provide stable regression estimates in Model 2.

Incidence of moderate or severe psychological distress was 33.3% (Table 2, 3). Across the sample, participants reported a median of 10 poor mental health days (IQR: 20) in the last 30 days. Respondents 25–34 years reported a median of 15 poor mental health days (IQR: 14) in the last 30 days, paralleling responses from adults ages 35–44 years (median: 15 days, IQR: 18), indicating that the majority of younger adult respondents experienced moderate or severe psychological distress. Median days of poor mental health were lower among adults ages 45–64 (median: 10; IQR: 20), and older adults ≥65 years (median: 0; IQR: 12).

### SGM respondents

Prevalence of psychological distress among individuals identifying as LGB + was 45.5%, with more than 50% of individuals identified as bisexual (51.9%), 37.0% of individuals identified as lesbian or gay, and 29.9% identified as heterosexual experiencing this outcome (Table 2, 3). Nearly 50% of individuals reporting other sexual orientations (asexual, questioning, or other) experienced psychological distress (48.6%). Prevalence of psychological distress among female respondents was 33.3% and 24.4% among male identified respondents. Nonbinary respondents and individuals reporting transgender experience had the highest prevalence of psychological distress among identity groups, with 64.8% and 60.4% experiencing this outcome.

**Fig 1:**
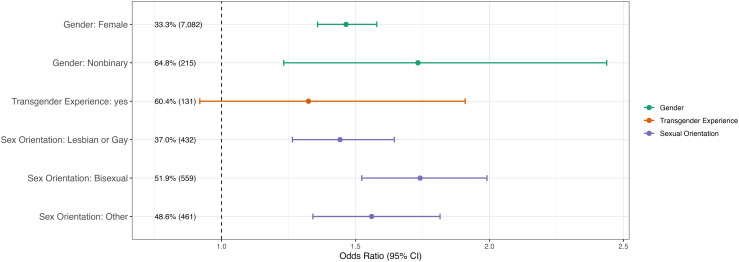
Adjusted odds ratio (95% CI) of psychological distress among sexual and gender diverse individuals in Massachusetts, Fall 2020. Estimates derived from Model 2, which includes more detailed identity variables with the following referent populations: male as the referent category for female and non-binary identities; cisgender as the referent for transgender experience; and heterosexual/straight as the referent for lesbian or gay, bisexual, and other sexual identities. Prevalence % (n) shown for each group.

In Model 1, individuals who reported LGB+ identity experienced 83% increased odds of psychological distress compared to heterosexual respondents (OR: 1.83 (1.34, 2.50; p = 0.0001)) (Table 2, 3). TNB individuals also reported significantly elevated odds of psychological distress (OR: 1.57 (1.21, 2.40; p = 0.0008). In Model 2, respondents identifying as lesbian or gay (OR: 1.44 (1.34, 2.50); p < 0.0001), bisexual (OR: 1.74 (1.52, 1.99); p < 0.0001), nonbinary (OR: 1.73 (1.23, 2.44) (p = 0.002), and female (OR: 1.46 (1.36, 1.58); p < 0.0001) experienced elevated odds of psychological distress compared to referents (Table 2, 3; Fig 1).

### Racial and ethnic identity

Prevalence of psychological distress was highest among respondents identifying as American Indian/Alaska Native (AI/AN) (40.4%) and multiracial NH (40.2%). In Model 1, respondents of color reported lower odds of psychological distress compared to white referents (OR: 0.68 (0.51, 0.91); p = 0.01) (Table 2, 3). In Model 2, Hispanic/Latinx and Black NH-identified respondents experienced 10–20% reduced odds of psychological distress respectively (OR 0.75 (0.67, 0.84); p < 0.0001); (OR: 0.75 (0.63, 0.88); p < 0.0001) compared to white respondents (Table 2, 3). Odds of psychological distress were 40% lower among Asian NH-identified respondents compared to white respondents (OR: 0.61 (0.51, 0.73); p < 0.0001). We observed marginally elevated odds of psychological distress among AI/AN-identified and multiracial NH respondents although neither association was statistically significant (Table 2, 3, Fig 1).

In intersectional analyses, we observed reduced odds of psychological distress among LGB+ respondents of color (OR: 1.03 (0.82, 1.29); p = 0.83) and TNB respondents of color (OR: 1.27 (0.73, 2.20); p = 0.39) compared to LGB+ and TNB estimates alone although neither interaction was statistically significant.

## Discussion

We observed elevated odds of psychological distress among SGM individuals in Massachusetts during the first year of the COVID-19 pandemic, with notably elevated odds of this outcome among the LGB + -identified group, respondents who identify as bisexual, and those who identify as non-binary compared to referents. Some racial and ethnic groups experienced reduced odds of reported psychological distress, including those identifying as Hispanic/Latinx, Black NH, and Asian NH, compared to White NH respondents. We conclude overall that SGM individuals experienced greater risk of psychological distress during this early pandemic period, with those holding certain identities having higher odds of this outcome compared to others.

We observed reduced odds of psychological distress among some but not all communities of color during the early pandemic period in Massachusetts. This is consistent with national trends that show reduced prevalence of poor mental health outcomes among certain communities of color [40]. Cultural norms around mental health, social stigma, and language used in data collection instruments may contribute to underreporting [41]. These factors may indicate that our estimates of poor mental health are underestimated among people of color completing the survey

Beyond potential underreporting, cultural stigma surrounding mental health, language barriers, and limited access to culturally competent care may also contribute to differential reporting and unmet need among communities of color. These factors can obscure the true burden of distress and highlight the importance of improving inclusivity in survey design, outreach, and data collection to better capture experiences across diverse cultural groups. Expanded representation in future surveillance and research is essential to inform equitable resource allocation.

Overall, we did not observe meaningfully increased odds of psychological distress among those with multiple marginalized identities (here SGM individuals of color). This heterogeneity of experience and/or reporting may be reflected in the trends presented for intersectional analyses. In intersectional analyses, non-significant trends showed reduced odds of psychological distress among LGB+ and TNB respondents of color compared to LGB+ and TNB estimates alone. Possible explanations include cultural or community coping mechanisms, resilience built through navigating multiple marginalized identities, and differences in help-seeking or mental health labeling that reduce reporting of distress. Alternatively, these findings may reflect selection bias, with SGM individuals of color experiencing the greatest stressors being less likely to participate in online surveys, be connected to organization that helped disseminate the survey, or to disclose their identities, leading to underestimation of distress in these groups. It is also possible that this is due to differential experience or reporting among respondents of color overall, or that the heterogeneity of both SGM and people of color is masking increased odds for some intersectional identities.

We observed fewer poor mental health days among older adults compared to younger age groups. Age-related differences in emotional regulation, coping, and exposure to pandemic-related stressors may contribute: older adults often report greater emotional stability and employ more adaptive regulation strategies, including reappraisal and selective focus on positive experiences [42–44]. Older adults may prioritize emotionally meaningful goals and positive affect, which may mitigate distress even under stressful conditions [45]. It remains possible as well that these findings are due to age-associated stigma in reporting mental health outcomes.

### Strengths of this analysis

The CCIS study was strengthened by a survey tool that intentionally and inclusively queried gender identity and sexual orientation and a sampling strategy that intentionally reached SGM communities and communities of color. While the CCIS study did not achieve a sample fully representative of the MA population, specifically around gender and education, the integration of a propensity score weighting scheme allowed us to address this challenge in analyses. Additionally, analyses comparing outcomes among combined and disaggregated SGM identities highlighted heterogeneity amongst groups traditional grouped in public health analyses. However, despite our interest in intersectionality of sexual orientation, gender identity, race, and ethnicity identities using disaggregated data, counts were too small in many paired interactions for meaningful analysis, despite the overall size of the CCIS sample.

### Limitations

Other limitations to this study include selection bias related to CCIS sampling methodology. Those with mental health disorders may have been less likely to participate in the survey and this could have disproportionately impacted participation by individuals with certain identities [46]. The CCIS used a snowball sampling strategy to engage populations often missed in surveillance, including SGM and racial or ethnic minority communities. While effective for reaching priority groups, this nonprobability approach may have preferentially captured individuals with stronger community or organizational ties, potentially excluding those with limited social connectedness or internet access. As a result, psychological distress could be underestimated among more isolated SGM respondents, and findings may not fully generalize across the broader population, despite weighting adjustments. This non-response bias may particularly impact intersectional analyses and interpretation due to smaller sample sizes for intersectional groups. We reiterate as well that this study was conducted in the fall of 2020 and may not be fully generalizable to other time periods, given the unique pandemic experience of that time. Additionally, the study was cross-sectional, and we are unable to distill trends over time.

### Implications

Our findings support a growing literature on mental health disparities experienced by SGM individuals during the COVID-19 pandemic. Notably, some research indicates improved mental health outcomes over time during the pandemic among SGM [47], and this is an area of future interest. While understanding the specific causes of this disparity are beyond the scope of the current study, efforts to promote public health policy and practice change to address root causes of health inequities, bolster social supports, and promote harm-reduction practices may be particularly efficacious in the context of SGM communities and to reduce the pandemic-associated mental health inequities. In particular, our work supports efforts to address structural drivers of mental health inequities, including expanded access to culturally competent and affirming mental health services, supporting telehealth and community- or peer-led programs that reach SGM individuals and communities of color, and strengthening data systems to routinely collect inclusive information on sexual orientation, gender identity, and race/ethnicity.

Further research on the effects of intersectionality, especially for SGM of color, is needed to distill the impact of multiple identities on mental health during this particularly challenging time and ensure that public health policy and practice is adapted to meet the needs of those holding multiple marginalized identities. Given the limitations of survey data, qualitative data collection among these communities would complement quantitative data and add an important and missing component to our understanding of the mental health experiences and needs of all Massachusetts residents.

## Conclusion

In this population-based study of Massachusetts residents during the early COVID-19 pandemic, sexual and gender minority (SGM) individuals experienced substantially higher odds of moderate or severe psychological distress compared to cisgender heterosexual peers, with the greatest burden among bisexual, nonbinary, and transgender respondents. These findings underscore persistent inequities in mental health risk. Mixed-methods research may complement our approaches to understand the reasons behind these inequitites and inform observations around intersectional identities. Public health action should focus on expanding access to affirming mental health services, strengthening inclusive data collection on sexual orientation and gender identity, and addressing structural barriers such as stigma, discrimination, and limits to gender-affirming care that exacerbate distress during crises.

## Supporting information

S1 Fig Diagram of propensity score modeling and merging of scores across two models. We combine propensity score vectors p1 and p2 in (C) depending upon CCIS gender category as categories 3 and 4 do not appear in PUMS.
(TIFF)

S1 Table Full multivariable logistic regression model (Model 1) predicting frequent psychological distress, Massachusetts, Fall 2020 (n = 26,889). (DOCX)

S2 Table Full multivariable logistic regression model (Model 2) predicting frequent psychological distress, Massachusetts, Fall 2020 (n = 26,889). (DOCX)
