# Supplementary figures and images for: Mental health inequities affecting sexual and gender diverse individuals during the early COVID-19 period in Massachusetts
Source: PLOS Ment Health. 2025 Dec 19;2(12):e0000341. doi: 10.1371/journal.pmen.0000341 (PMC12798262; doi:10.1371/journal.pmen.0000341)

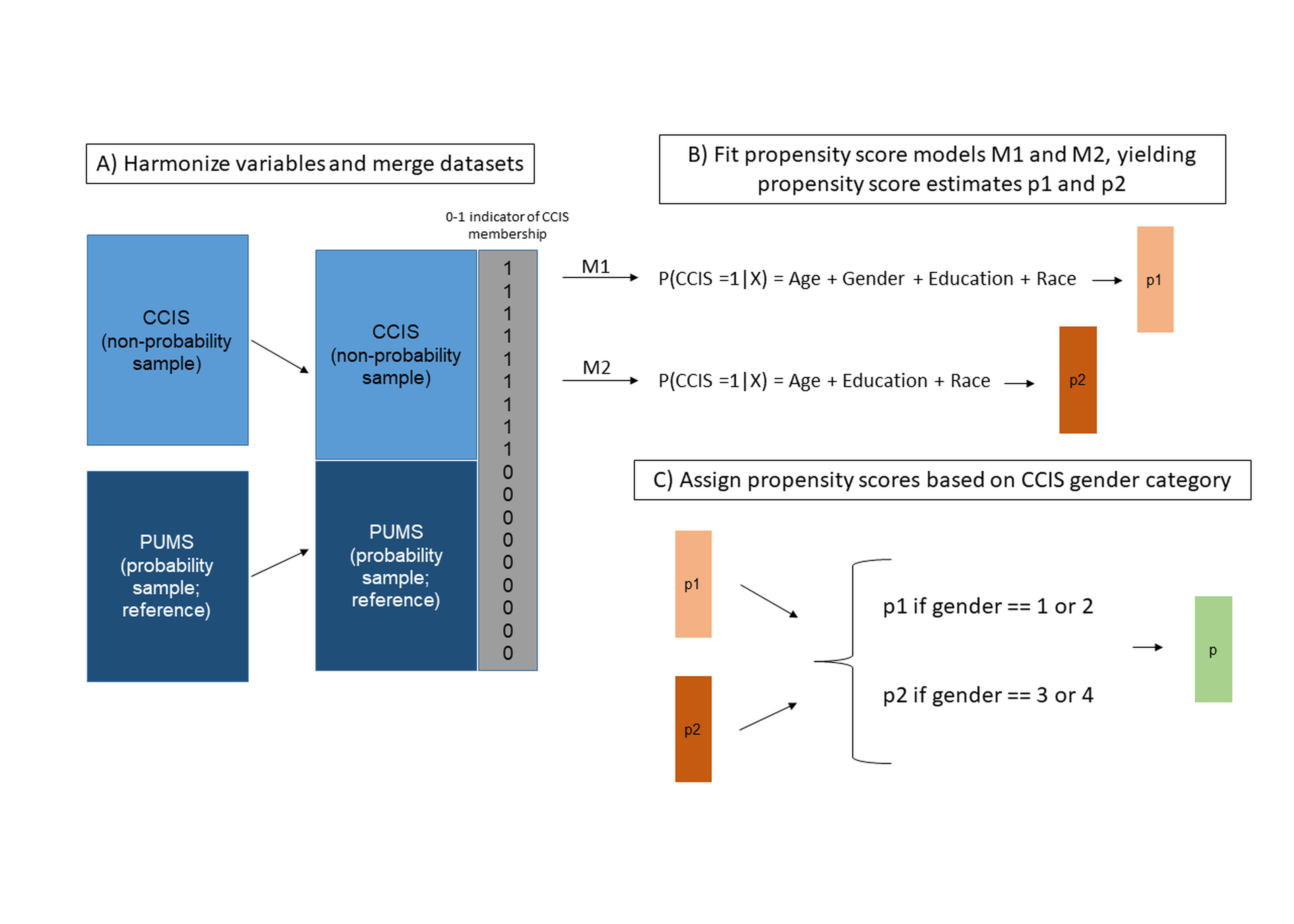

Supplement: S1 Fig — We combine propensity score vectors p1 and p2 in (C) depending upon CCIS gender category as categories 3 and 4 do not appear in PUMS. (TIFF) [file pmen.0000341.s001.tiff]
