# Supplementary material for: Mental health inequities affecting sexual and gender diverse individuals during the early COVID-19 period in Massachusetts
Source: PLOS Ment Health. 2025 Dec 19;2(12):e0000341. doi: 10.1371/journal.pmen.0000341 (PMC12798262; doi:10.1371/journal.pmen.0000341)
Supplement: S1 Table — (DOCX) [file pmen.0000341.s002.docx]

S1 Table: Full multivariable logistic regression model (Model 1) predicting frequent psychological distress, Massachusetts, Fall 2020 (n = 26,889)

| Section | Predictor | OR (95% CI) | p-value |
| --- | --- | --- | --- |
| Other covariates | (Intercept) | 1.88 (1.52,2.32) | 0.000 |
| Sexual orientation | LGB | 1.83 (1.34,2.50) | 0.000 |
| Gender / Transgender | Non-cisgender | 1.57 (1.21,2.04) | 0.001 |
| Race and ethnicity | People of color | 0.68 (0.51,0.92) | 0.011 |
| Age | Age (years) | 0.97 (0.97,0.98) | 0.000 |
| Income | Income < 35K | 0.69 (0.51,0.94) | 0.019 |
| Income | Income > 100K | 0.81 (0.64,1.03) | 0.084 |
| Education | College education | 0.84 (0.79,0.91) | 0.000 |
| Region | Eastern Mass | 1.11 (1.01,1.21) | 0.024 |
| Region | Southern Mass | 1.12 (0.93,1.35) | 0.223 |
| Region | Western Mass | 1.18 (1.06,1.31) | 0.003 |
| COVID context | Town-level COVID high | 0.97 (0.92,1.03) | 0.379 |
| Employment | Retired | 0.74 (0.65,0.84) | 0.000 |
| Employment | Unemployed | 1.74 (1.56,1.95) | 0.000 |
| Interactions | LGB ×People of color | 1.05 (0.83,1.31) | 0.695 |
| Interactions | Non-cisgender × People of color | 1.32 (0.75,2.34) | 0.342 |
| Interactions | People of color × age | 1.00 (1.00,1.01) | 0.350 |
| Interactions | LGB × age | 1.00 (0.99,1.00) | 0.158 |
| Interactions | LGB × Income< 35K | 1.11 (0.87,1.42) | 0.387 |
| Interactions | LGB × Income >100K | 1.02 (0.85,1.21) | 0.863 |
| Interactions | age × Income< 35K | 1.01 (1.01,1.02) | 0.000 |
| Interactions | age ×Income >100K | 1.00 (0.99,1.00) | 0.311 |
