# Supplementary material for: Mental health inequities affecting sexual and gender diverse individuals during the early COVID-19 period in Massachusetts
Source: PLOS Ment Health. 2025 Dec 19;2(12):e0000341. doi: 10.1371/journal.pmen.0000341 (PMC12798262; doi:10.1371/journal.pmen.0000341)
Supplement: S2 Table — (DOCX) [file pmen.0000341.s003.docx]

S2 Table: Full multivariable logistic regression model (Model 2) predicting frequent psychological distress, Massachusetts, Fall 2020 (n = 26,889)

| **Section** | **Predictor** | **OR (95% CI)** | **p-value** |
| --- | --- | --- | --- |
| Other covariates | (Intercept) | 1.25 (1.05,1.49) | 0.014 |
| Sexual orientation | Bisexual | 1.74 (1.52,1.99) | 0.000 |
| Sexual orientation | Lesbian or Gay | 1.44 (1.26,1.64) | 0.000 |
| Sexual orientation | Other | 1.56 (1.34,1.82) | 0.000 |
| Gender / Transgender | Female | 1.46 (1.36,1.58) | 0.000 |
| Gender / Transgender | Nonbinary | 1.73 (1.23,2.44) | 0.002 |
| Gender / Transgender | Transgender | 1.32 (0.92,1.91) | 0.132 |
| Race / Ethnicity | AI/AN | 1.18 (0.89,1.55) | 0.246 |
| Race / Ethnicity | Asian NH | 0.61 (0.51,0.73) | 0.000 |
| Race / Ethnicity | Black NH | 0.75 (0.63,0.88) | 0.001 |
| Race / Ethnicity | Hispanic | 0.75 (0.67,0.84) | 0.000 |
| Race / Ethnicity | Multiracial | 1.11 (0.88,1.40) | 0.377 |
| Race / Ethnicity | Other Race NH | 1.14 (0.82,1.56) | 0.431 |
| Age | Age (years) | 0.98 (0.97,0.98) | 0.000 |
| Income | Income < 35K | 1.35 (1.23,1.49) | 0.000 |
| Income | Income > 100K | 0.74 (0.70,0.79) | 0.000 |
| Education | College education | 0.85 (0.79,0.91) | 0.000 |
| Region | Eastern Mass | 1.11 (1.01,1.21) | 0.027 |
| Region | Southern Mass | 1.10 (0.91,1.32) | 0.338 |
| Region | Western Mass | 1.16 (1.04,1.29) | 0.008 |
| COVID context | Town-level COVID high | 0.99 (0.93,1.05) | 0.658 |
| Employment | Retired | 0.78 (0.69,0.88) | 0.000 |
| Employment | Unemployed | 1.75 (1.56,1.96) | 0.000 |
